# Let the Veterinary Organizations Move in the Matter

**Published:** 1901-02

**Authors:** 


### LET THE VETERINARY ORGANIZATIONS MOVE IN THE MATTER.

FOLLOWING our meeting in Nashville, September 1897, the establishment there of a veterinary college with scarcely any equipment of any kind seemed an added menace to higher veterinary education; but the attitude of the profession, strengthened and sustained by our convention in the capital city of the State, restrained every self-respecting veterinarian from allegiance to or connection with this new institution. In want of building equipment, lacking in laboratory adjuncts, weak in corps of instructors, and with little to commend it in any direction as a source of higher veterinary education, the people of the "Sunny South" have not been slow to measure it at its worth and what it has lacked in every way needful for college work. It has more than felt the hunger for students—some three students the first year, and it is said to have but two scholars this year. Such may be said to be the logical fruits of a seed-planting in new territory, and we must be up and moving at all times to restrain, prevent, crush out, starve out, if necessary, all such institutions as parade under such false colors.

---

WE desire to call especial attention of our readers to the coming annual meeting of the Pennsylvania State Veterinary Medical Association.

This organization, one of the strongest State Associations in existence, always attracts attention by the breadth and value of its work. Again, this year, it will conduct a post-graduate course for all veterinarians in the State. A splendid programme is assured, excellent clinics will be provided, and in addition thereto the JOURNAL OF COMPARATIVE MEDICINE AND VETERINARY ARCHIVES will tender a dinner to all the visiting delegates, members of the Association, and profession in attendance.

---

THE various State Legislatures now in session should be watched by the veterinary profession with a vigilant eye for legislation that may be of the utmost importance to the profession's well-being as well as for the good of every community.

